# Technical Report: Percutaneous Reductions on Gartland Type III Supracondylar Fractures

**DOI:** 10.7759/cureus.26202

**Published:** 2022-06-22

**Authors:** Rafaela M Gonçalves, Mariana O Lobo, Flávio E Azevedo, Felipe M Braga, Anderson Freitas

**Affiliations:** 1 Orthopedics and Traumatology, Hospital Regional of Gama, Brasília, BRA; 2 Orthopedics and Traumatology, Hospital Regional of Gama, Brasilia, BRA

**Keywords:** elbow fracture, pediatric surgery, pediatric trauma, closed reduction, supracondylar fractures

## Abstract

Various surgical treatments for supracondylar fracture in children remain the motive of research and study. A common factor in each technique is anatomic reduction, which is critical for the excellent recovery of the fracture. Anatomic reduction is preferably realized through closed and percutaneous approaches. This study aimed to present a closed percutaneous technique to treat Gartland III supracondylar fractures to aggregate a facilitating factor in the surgical approach to this condition. Our technique was applied to surgical patients in orthopedic and traumatology emergency care and illustrated by a synthetic elbow model, including soft tissue and intraoperative images.

## Introduction

Supracondylar fractures in children comprise the most considerable portion of surgical treatments in the pediatric population, representing 3% to 15% of all childhood fractures [1,2]. In stable fractures, the preferred treatment method for displaced supracondylar fractures is closed reduction and percutaneous pinning [3,4]. However, the preferred method can be challenging to perform due to edema, local hematoma, instability, and degree of deviation [1]. Closed reduction can be accomplished using the joystick technique in complex cases, preserving the posterior periosteum. However, a new approach to managing percutaneous reductions on supracondylar fractures can offer a straightforward approach and increase the portfolio of surgical treatments to address this pathology [5,6].

## Technical report

**Figure 1:**
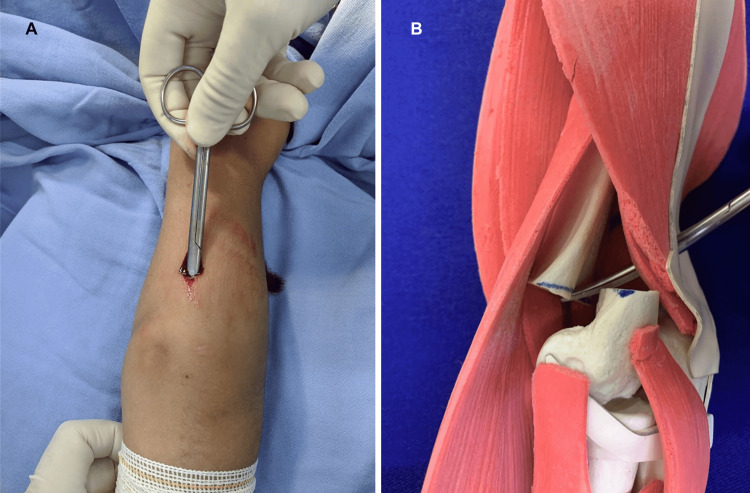
(A) Intraoperative image of an incision on the posterior surface of the arm, at the fracture site, over fluoroscopy image intensifier. (B) Synthetic model including tissue: illustration of the internal positioning of scissor trespassing the supracondylar fracture site.

In posteriorly displaced fractures, the patient is in a prone position with the elbow resting on the image intensifier of the fluoroscopy machine. Exsanguination was not performed in our cases, but it can be used according to the surgeon's preference. We start by trying to obtain bone fragment alignment through closed reduction. If attempts of closed reduction fail, we then realize longitudinal traction of the affected limb, with the elbow in extension, to obtain a slight alignment of the fractured fragments, correcting the deviation on the coronal plane. Rotational misplacements are harder to rectify, but the technique helps to achieve its alignment. We execute a small incision on the posterior surface of the arm at the fracture level via a blunt trans-tricipital dissection with a blunt instrument (Kelly clamp or scissors, both with curved tips), reaching the fracture site that will be breached until the tip of the instrument hits the anterior edge of the proximal humerus fragment (Figures 1A, 1B). This approach does not involve any muscular plane of the posterior compartment. The blunt instrument must be introduced carefully, avoiding deep advances on the anterior edge of the proximal humerus fragment, to minimize the risks of neurovascular injuries.

**Figure 2:**
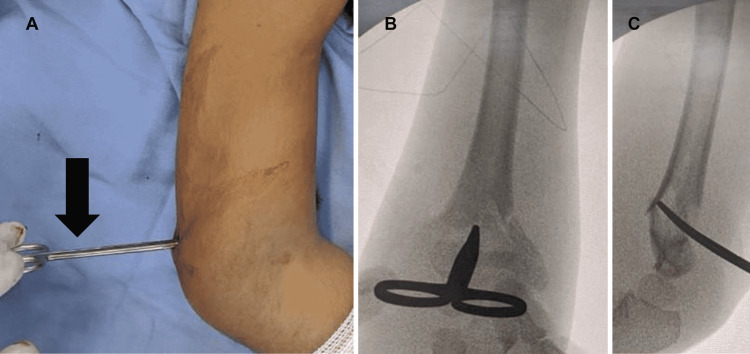
(A) Intraoperative image demonstrating the scissor movement, already at the fracture site. (B) Fluoroscopic image in anteroposterior view demonstrating the reduction during the maneuver. (C) Fluoroscopic image in lateral view demonstrating the reduction during the maneuver.

**Figure 3:**
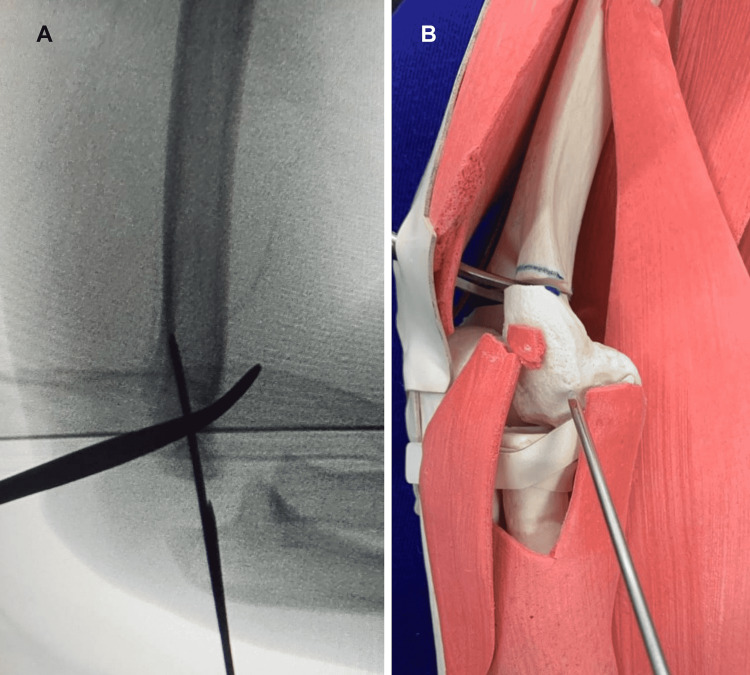
(A) Fluoroscopic image of a lateral view of the elbow demonstrating adequate reduction with the scissor positioned to secure its maintenance during lateral wall fixation. (B) Image of the synthetic model of the elbow, including soft tissue showing the surgical moment of the lateral wall fixation.

Moving the surgical instrument on the longitudinal axis of the arm in the distal-proximal direction, the posterior part of the instrument will be pushing and anteriorizing the distal fracture fragment (distal humerus), causing the Kapandji effect of reduction (Figures 2A-2C) [7], allowing fragment movement and the reduction of the supracondylar humerus fracture in a less traumatic form. After radioscopy evaluation shows a good reduction pattern, the surgeon can execute the pinning in the desired way, starting from the lateral column (Figures 3A, 3B) according to the required quantity of wires. It should be noted that the technique does not prevent ulnar nerve injury, especially when pinning the medial column.

**Figure 4:**
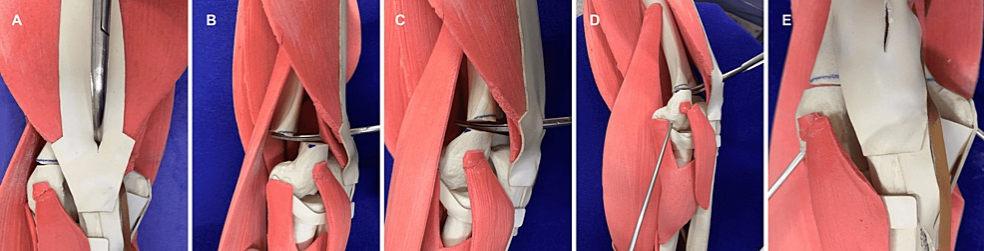
Image progression of the elbow's synthetic model, including soft tissue, demonstrates each step of the technique (A-E).

**Figure 5:**
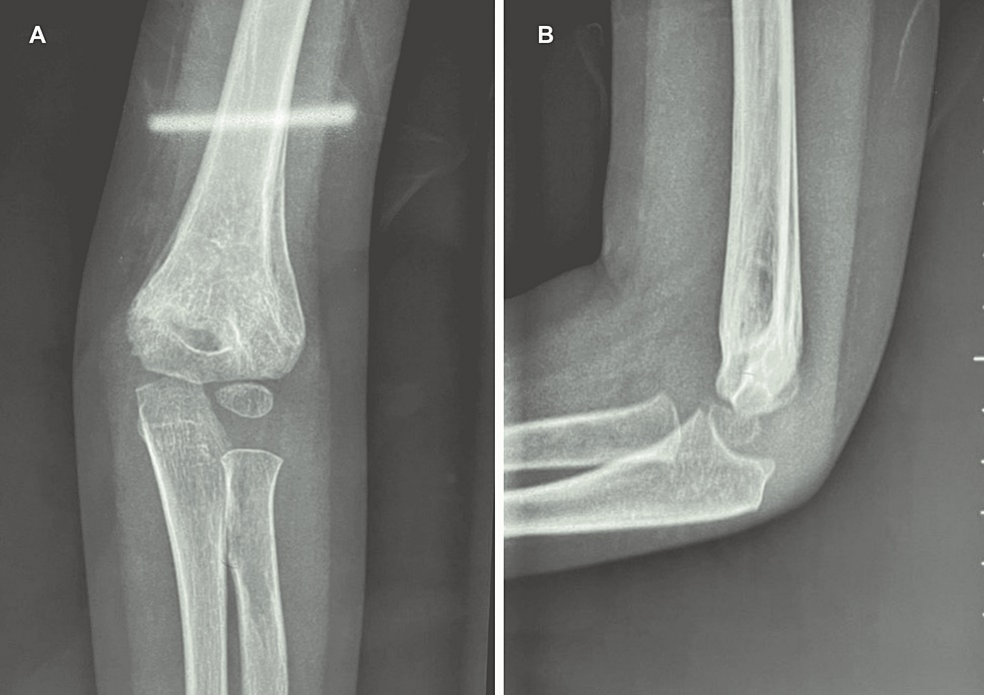
Postoperative x-ray in the anteroposterior view (A) and lateral view (B) of the elbow.

For the crossed wires technique, the instrument should be kept to better control the reduction and its maintenance during the position shifting of the elbow. Another method is temporarily securing more than one wire in the lateral column to avoid rotational loss of the reduction. This way, the immediate postoperative fixation will be done with less soft tissue manipulation, less structural damage, and better preservation of the local biology (Figures 4A-4E, 5A, 5B).

## Discussion

“Give me a lever and a place to stand, and I will move the earth,” the famous phrase of the Greek mathematician Archimedes de Siracusa (287-212 a.C) [8] that not only conveys the concept of balance in the law of levers but also highlights the conception of the technique described in this review: the principle of intrafocal fixation described by Kapandji [7]. Kirschner wires inserted directly on the fracture line is one of many orthopedics techniques that apply the law of levers, providing energy to obtain anatomic reduction and minimizing added injuries resulting from the reduction procedure. 

On the supracondylar fracture, open reduction and percutaneous pinning are associated with a higher risk of complications, such as joint stiffness, neuropraxia, and heterotopic ossification [3,6]. Therefore, in the absence of exposed, highly deviated, or irreducible fractures and neurovascular injuries on Gartland Type III fractures, open reduction, and percutaneous pinning are the standard treatment choice. The difficulties related to the reduction procedure are mitigated by the technique described here and the joystick technique (Table 1) [1,5]. Of the four cases where the technique was applied, one was unsuccessful because the distal fragment kept getting exaggeratedly anteriorized during its manipulation, requiring open reduction. We noticed the violation of the posterior periosteum, which was then treated as a Gartland type IV supracondylar fracture.

**Table 1:** Gartland fracture classification

Type I	Nondisplaced
Type II	Intact posterior cortex, hinged in extension
Type IIA	Not displaced on the coronal image
Type IIB	Presence of some degree of rotational or translational displacement
Type III	Completely displaced: proximal and distal fragments without communication
Type IV	Multidirectional instability with circumferential periosteal disruption. Instability in flexion and extension can be verified through fluoroscopic images during the surgical procedure.

Even though the technique described demonstrated effectiveness, it is necessary for its ratification through epidemiological studies and clinical trials.

## Conclusions

We described a technique facilitating closed reductions of Gartland type III supracondylar fractures. Our technique is indicated in cases of multiple failed closed reduction attempts or misaligned closed reductions, and should be considered before open reduction and internal fixation. To obtain the desired alignment on the coronal plane, traction of the affected limb with the elbow in extension is sufficient for its achievement. This technique is not exempt from neurovascular risks. However, they are greatly minimized if introducing the blunt instrument carefully and not breach the anterior edge of the proximal humerus fragment. This technical report emphasizes the importance of periosteum integrity related to the employment of the technique. We also call attention to the urgency of the treatment of such fractures and the benefits of a closed procedure, minimizing the risks of neurovascular injuries and soft tissue damage.
